# A Search for Energy Minimized Sequences of Proteins

**DOI:** 10.1371/journal.pone.0006684

**Published:** 2009-08-19

**Authors:** Anupam Nath Jha, G. K. Ananthasuresh, Saraswathi Vishveshwara

**Affiliations:** 1 Molecular Biophysics Unit, Indian Institute of Science, Bangalore, India; 2 Department of Mechanical Engineering, Indian Institute of Science, Bangalore, India; Naval Research Laboratory, United States of America

## Abstract

In this paper, we present numerical evidence that supports the notion of minimization in the sequence space of proteins for a target conformation. We use the conformations of the real proteins in the Protein Data Bank (PDB) and present computationally efficient methods to identify the sequences with minimum energy. We use edge-weighted connectivity graph for ranking the residue sites with reduced amino acid alphabet and then use continuous optimization to obtain the energy-minimizing sequences. Our methods enable the computation of a lower bound as well as a tight upper bound for the energy of a given conformation. We validate our results by using three different inter-residue energy matrices for five proteins from protein data bank (PDB), and by comparing our energy-minimizing sequences with 80 million diverse sequences that are generated based on different considerations in each case. When we submitted some of our chosen energy-minimizing sequences to Basic Local Alignment Search Tool (BLAST), we obtained some sequences from non-redundant protein sequence database that are similar to ours with an E-value of the order of 10^-7^. In summary, we conclude that proteins show a trend towards minimizing energy in the sequence space but do not seem to adopt the global energy-minimizing sequence. The reason for this could be either that the existing energy matrices are not able to accurately represent the inter-residue interactions in the context of the protein environment or that Nature does not push the optimization in the sequence space, once it is able to perform the function.

## Introduction

Optimization is inherent to proteins, which are linear chains of amino acid residues. Anfinsen's hypothesis [1] is one example of minimization of free energy in the conformation space, which is the collection of three dimensional folded configurations of a protein chain. This enables first principles approaches to protein structure prediction [2], [3]. Protein structure prediction involves three steps. The first is to identify the segments of the protein chain that form secondary structures, namely, alpha helices and beta strands formed due to Hydrogen bonds between the backbone carbonyl oxygen and the peptide nitrogen atoms. The second step is to identify the pairs of beta strands which form hydrogen-bonded beta sheets in parallel or anti-parallel form. The third step involves the identification of inter-residue interactions which optimally orients the secondary structures, linked by loops.

The complete set of possible sequences for a given conformation is called the sequence space. For a *N*-residue chain, there will be 20*^N^* sequences in the sequence space. Protein design, therefore, implies identifying the sequences that will fold to a target conformation. The challenge however is to ensure that the selected sequence indeed prefers the desired conformation. Thus, the larger problem involves the search both in the sequence and in the conformational space. In this paper we are limiting our search for sequence space, which is not studied as extensively as the search in the conformational space. There is no established guiding principle or hypothesis for searching the protein sequence space. However, it is a common practice to minimize the energy in the sequence space [4]–[8]. These approaches assume that there is a notion of minimization in the sequence space. Specifically, when a conformation is chosen and we need to find sequences that are likely to fold to that conformation, often energy-minimizing sequences are searched for the chosen conformation. The motivation for the present work is the development of a computationally efficient method to generate sequences that minimize the energy for a given conformation.

A search for new sequences by re-design of known proteins as well as de novo protein design is beneficial. Proteins can be engineered to have certain unusual and favorable properties. For example, Baker's group [9], [10] found that computer-generated proteins folded much faster than the wild types. Proteins are designed to have new metal binding sites on a backbone template that is not known to have such binding sites [11]. The stability of the engineered proteins can be enhanced [12]. De novo protein design methods are useful for generating sequences with better capabilities to fight diseases as shown by the enhanced the antimicrobial property of 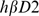, a 41-residue peptide [13]. Improved specificity is also possible as demonstrated [14] in the case of myoglobin family.

De novo protein design necessarily requires a search in the sequence space. Although there is no guiding principle, many experimental and computational approaches have been made. Experimental approaches such as mutagenesis, rational design, and directed evolution sample up to a few million sequences [2], [15], which is far too small compared to the possible number of sequences. Computational approaches can consider much larger number of sequences but the problem is overwhelming even for modern computing power. Self-consistent mean field theory [16]–[18] and dead-end elimination [19], [20] approaches have been attempted to search the sequence space. These approaches consider the side chain rotamer configurations, which is an added dimension to the search in the sequence space. Such approaches aim to achieve optimal packing that avoids steric hindrances and have had some success. Genetic algorithms [21] and Monte Carlo simulations [22] have been attempted to sample a large space of sequences. Assigning probabilities for each amino acid to occupy every residue site is another approach that has been followed for optimization in the sequence space [23], [24]. Levitt and co-workers [14], [25] emphasize that the both the sequence and conformation spaces must be searched sequentially or simultaneously to retain the specificity of proteins. Another interesting observation made by Levitt's group is that it is imperative to fix the composition of the twenty amino acid residues in a protein in the sequence space search to retain the specificity [26], [27]. As one can expect, in the absence of such a constraint, there will be a tendency towards lowest-energy amino acid occupying all the residue sites. Similar feature was also noted by Dill's group [28] in the context of HP models. Some recent work has considered flexible target backbone conformations [29], [30] in order to achieve the specificity.

The goal of present work is to explore efficient methods of energy-minimized sequences for a target protein. Our earlier work used graph theory [31] and continuous modeling of the sequence space to generate energy-minimizing sequences [32], [33] in the HP model. In this work, we present an improved method that combines these approaches to find the lower bound on the energy for a target conformation. Furthermore, sequences are searched in the reduced (five) alphabets model [34] of amino acids. While HP model is too simplistic, considering all 20 amino acids is computationally intractable. Hence, several efforts have been made to group amino acids into a number between 2 and 20. For this, a number of criteria can be used. For example, Venkatarajan and Braun [35] used 237 properties to group amino acids. Recently, our group used metric multi-dimensional scaling (MMDS) to develop a technique to group 20 amino acids into any number of required groups [36]. In that work, we showed that group with five alphabets is optimal. Hence, in this work, we are using five-grouping as a first step in our procedure. Later on, we do consider all 20 amino acids so that no generality is lost.

This new method, unlike others presented in the literature, takes only a few minutes of computations on a single-processor P4 desktop computer. By using this technique, we have shown that a large number of sequences with energies better than the native sequence can be generated. Additionally we also show that the energy of the native sequence is much lower, in comparison to the energies of the random sequences threaded on the target conformation. This lends support to the notion of minimization of the energy in the sequence space. We further demonstrate that designing sequences by constraining a fraction of the amino acids to their position in the native sequence will yield sequences which are similar to the native one, at the same time shifts the mean energy towards the native sequence. Our results hold well for three different inter-residue pair-wise energy models and for five proteins of different folds. Thus, our results not only present concrete evidence to minimization in the sequence space (but not to the global minimum) but also show promise for a computationally efficient method for de novo protein design.

## Results

**Figure 1:**
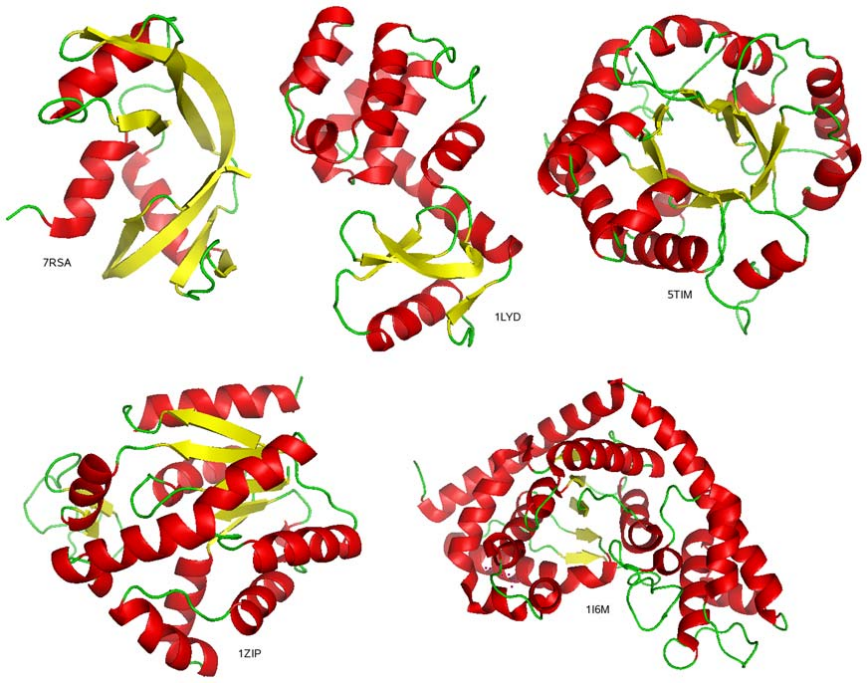
Proteins structures considered in the current study. PDB codes are: 7RSA, 1LYD, 5TIM, 1ZIP, and 1I6M.

We considered five proteins from the Protein Data Bank [37]: Ribonuclease A (7RSA), T4-Lysozyme (1LYD), Bacillus Stearothermophilus Adenylate Kinase (1ZIP), Triosephosphate Isomerase (5TIM), and Tryptophanyl-trna Synthetase (1I6M), whose *C_α_* backbone conformations are shown in Figure 1.

We have generated eight different sets of sequences (each of them containing 10 million sequences) by applying different criteria (given in Table 1 and explained in Methods) for Ribonuclease A (7RSA) and T4-Lysozyme (1LYD). Since the trends were the same in these two, we generated only the 1^st^ and 5^th^ sets of sequences for the remaining three proteins. The energy distributions for all the cases are calculated for three different inter-residue energy matrices [38]–[40] but the results are presented only for MJ matrix [38] as discussed next.

**Table 1:** Generation of the eight sets of sequences from the native sequence.

Set No.	Method of generation*
*Random sequences*	
1	Completely random set
2	The conserved residues in the protein family (∼10%) are constrained in their structural position and others were randomly assigned
3	10% of the top ranked^a^ residues are constrained in their structural position and others were randomly filled
4	Random sequences with the constraint of arbitrary selected group of five alphabet (four amino acids to each)
*Designed sequences*	
5	Designed sequences based on ranking^a^ and reduced amino acid (five) alphabets^b^
6	Designed sequences with the conserved residues being constraint in their structural position
7	Designed sequences with the residues in the largest cluster^c^ being constrained
8	Designed sequences with 10% of the ranked^a^ residues are constrained in their structural position

* the residues composition all the generated sequences is the same as of the native sequence.

a topology based ranking scheme (Jha et al. 2007).

b reduced amino acid group of five alphabet (Luthra et al. 2007).

c largest cluster of side-chain based interacting amino-acids at I_min_ = 8% (Brinda and Vishveshwara 2005).

### (a) Energies of the designed and randomly generated sequences

**Figure 2:**
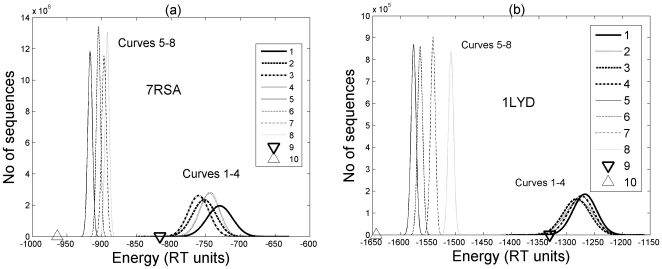
Energy profile of random and designed sequences. Energy distribution of set of random (curves 1–4) and designed (curves 5–8) sequences for 7RSA shown in Figure 2a and for 1LYd in Figure 2b. The triangle marker on the left indicates the lower bound energy while the inverted triangle marker shows the energy of the real sequence.

Ten million sequences were generated in each of the eight sets (see Table 1) for ribonuclease-A and lysozyme. They were threaded onto the native conformation and the energies of the protein structure of these sequences were evaluated by using pair-wise potentials as described in the Methods section. The results are presented in Figures 2a and 2b. As expected, the energies of the random sequences followed the Gaussian distribution indicated by curve 1 in Figures 2a–b. Interestingly we see that the energy of the native sequence (indicated by inverted triangle (▾)) is at the tail end of the randomly generated sequences. Indeed, the energy of the native sequence is at least one standard deviation lower than the mean energy of the random sequences in the Gaussian distribution curve. Curves 2, 3 and 4 correspond respectively to the randomly generated sequences by constraining some of the residues to their position on the basis of conservation, top ranks in the structure (ranking method described in the method section), and residues belonging to the same group (described in the Method section). The mean energies in these constrained cases are lower than that of the completely random set.

This means that any bias towards the native sequence decreases the energy and moves towards the energy of the native one. The fact that the energy of the native sequence lies at the tail end of the random sequences (40 million, as indicated by curves 1–4), clearly shows that the energy of the native sequence is minimized in the sequence space. This observation is consistent with what has been reported in the literature [29].

**Figure 3:**
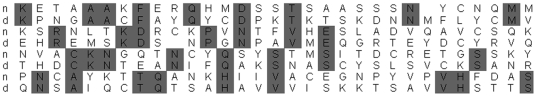
Sequence similarity between native and the designed sequence. The native (indicated with n) and one of the designed (indicated with d) sequences of Ribonuclease A (7RSA). There are 30% residues at the same positions in two sequences as shown above with shaded blocks around the single-letter codes of the amino acids.

Although we observe that the native sequences are optimized in the sequence space, we find many more sequences with much lower energies in a different part of the sequence space as shown by curves 5–8 in Figures 2a–b. Curve 5 in Figures 2a–b shows the energy distribution of 10 million sequences generated by our edge-weight ranking method that used five-alphabet amino acid grouping [34] (see Methods section). Curves 6–8 show the energy distributions of 10 million sequences each in which some functionally or structurally important sites were conserved in different ways (see Methods section). As can be seen in the figure, the mean energies of these 40 million designed sequences are much lower than those of the random sequences (curves 1–4) and interestingly also lower than the native sequence. The triangle marker (Δ) in Figures 2a–b shows the lower bound on the energy for the chosen conformation as obtained by our optimization method. This indicates that the native sequence is not going for the globally minimum energy in the sequence space. In fact we find that the lower bound energy is 8–20 standard deviations smaller than the mean energy of the designed sequences and the energy of the native sequence. This indicates that the native sequence does not adopt the global minimum in the sequence space. An equally important factor to notice is that any bias by way of conservation of selected sites (shown in curves 6–8) pushes the energy distribution towards the native sequence. This is significant because it enables us to study what criteria or biases would lead to sequences that resemble the native sequence. In fact, the sequence with the lower bound energy has around 30% residue at the identical positions (in case for 7RSA) as in the native sequence as shown in Figure 3.

**Figure 4:**
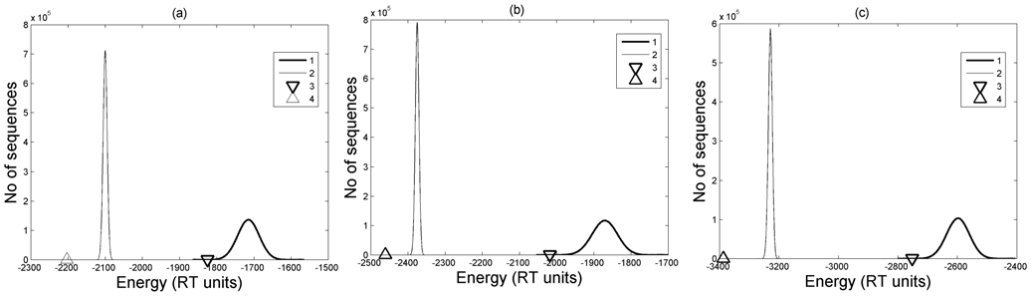
Energy profile of random and designed sequences. Energy distributions of random (curve 1) and designed (curves 2) sequences obtained for three proteins (a) 1ZIP, (b) 5TIM, and (c) 1I6M. The triangle marker (Δ) on the left indicates the lower bound energy while the inverted triangle marker on the right shows the energy of the native sequence. Notice that the energy of the native sequence is in between the mean energies of the random and designed sequences' energy distributions. The lower bound energy found by our method is much lower than the energy of the native sequence in all cases.

The same behavior that was explained above for Ribonuclease A and Lysozyme was observed for the other three proteins. Figures 4a-c show that the energy of the native sequence is always straddled between the energies of the random sequences and the designed sequences. The mean energy and the standard deviation for designed and random sequences for all the chosen proteins have been summarized in Table 2. It shows that the energy of the native sequence is at least one standard deviation lower than the mean energy of the generated random sequences whereas it is very high (in the range of 7–22 standard deviations) than the mean energy of the designed sequences. Another interesting point is that the standard deviation for the random sequences is much larger than of the designed sequences. It shows that the energies of the designed sequences are not widely spread like random sequences and the Gaussian distribution for them have a sharp peak. The same behavior was seen in all the five proteins that we considered here. The mean of the energy of the random sequences gives a tight upper bound on the energy for a given target conformation. Thus, we have been able to provide a lower and an upper bound for the energy of the sequences for a given protein.

**Table 2:** Standard deviation for the random and the designed sequences.

S. No.	PDB code	No. of residues	Native energy (*E_n_*)	Mean (*µ_rand_*) of random sequences	Std. dev. (*σ_rand_*) of random sequences	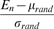 of random sequences	Mean (*µ_em_*) of designed sequences	Std. dev. (*σ_em_*) of designed sequences	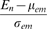 of designed sequences
1	7RSA	124	−816.02	−730	58.17	*−1.48*	−917.5	13.42	*7.56*
2	1LYD	164	−1331.5	−1285	78.38	*−0.59*	−1578	16.31	*15.11*
3	1ZIP	217	−1824.61	−1715	87.04	*−1.26*	−2102	19.2	*14.45*
4	5TIM	249	−2017.69	−1878	102.9	*−1.36*	−2378	16.31	*22.09*
5	1I6M	326	−2752.46	−2602	114.5	*−1.31*	−3232	24.97	*19.20*

We note that the above observations hold good for three different inter-residue energy matrices [38]–[40] that we used to test the consistency of our observations. It is clear that the method of evaluating energy has no significant influence on our observations.

### (b) Similarity between generated and existing sequences

The similarity between the native (7RSA and 1LYD) and the generated sequences were found by using NCBI BLAST program [41] for all sets of corresponding sequences. The sequence with the lowest E-value from each generated set was BLASTed against the NCBI non-redundant protein sequences database. No hits were obtained for the sequence obtained from the set, which was not biased even though it had 30% residues at the same position. The sequence from other three sets with 10% fixed residues (conserved in the family of that protein, components of largest connected cluster, or highly ranked; curves 6, 7, and 8 respectively in Figures 2 (a) and (b)) gave hits with E-value close to e^−07^. The top ten hits obtained for 7RSA and 1LYD are given in Table 3 and Table 4 respectively. These results indicate that retaining a small fraction (in this case 10%) of the residues at their original position gives rise to sequences closer to existing ones in the database.

**Table 3:** BLAST results for Ribonuclease A (7RSA) (sequence identity 33%).

Protein name*	PDB	Score	E-value
C[40,95]a Variant of Bovine Pancreatic Rnase A	1A5P	58.5	1e-07
Study of Reductive Unfolding Pathways of Rnase A (Y92g Mutant)	1YMW	57.4	3e-07
Crystal Structure of F120a Mutant of Bovine Pancreatic Rnase A	1EIC	57.4	3e-07
Structure of The P93g Variant of Rnase A	3RSP	57.4	3e-07
Crystal Structure of F120g Mutant of Bovine Pancreatic Rnase A	1EID	57.4	4e-07
Structure of A Synthetic, Non-Natural Analogue of Rnase A	2OQF	57.0	5e-07
Structure of A Cis-Proline (P114) to Glycine Variant of Rnase A	1KH8	57.0	5e-07
Crystal Structure of F120w Mutant of Bovine Pancreatic Rnase A	1EIE	57.0	5e-07
Thr45gly Variant of Rnase A	1C8W	57.0	5e-07
X-Ray Structure of Synthetic [d83a] Rnase A	2NUI	57.0	5e-07

* the length of submitted sequence and the alignment length are 124.

**Table 4:** BLAST results for T4-Lysozyme (1LYD) (sequence identity 30–35%).

Protein name*	PDB	Score	E-value
Alanine replacements within alpha- helix 126-134 of T4 Lysozyme	1L72	49.7	6e-05
N-Phenylglycinonitrile in complex with T4 Lysozyme	2RBN	49.7	7e-05
T4 Lysozyme mutant L99aM102Q	1LGU	49.7	7e-05
Site-Directed mutations of T4 Lysozyme	1L24	49.3	9e-05
Thr 157 to the thermodynamic stability of Phage T4 Lysozyme	1L12	47.8	2e-04
Alanine replacements within alpha- helix 126-134 of T4 Lysozyme	1L71	47.4	3e-04
The combination of point Mutations in T4 Lysozyme	189L	46.6	5e-04
Halide Binding Site to Bypass the 1000-atom limit to ab initio Structure Determination	1SWY	46.6	6e-04
Alpha-Helix Propensity within the context of a Folded Protein: Sites 44 and 131 in Bacteriophage	1DYE	46.6	6e-04
The Stability of T4 Lysozyme determined by directed Mutagenesis	1L38	46.6	7e-04

* the length of submitted sequence is 164 whereas the alignment is 102.

### (c) Contact performances in the designed sequences

To compare the distribution of different interacting amino acid pairs in random sequences and the designed sequences, we took about 10,000 sequences from each of the mid region of random, mid of the designed ones, and from the tail region of random sequences (close to native one). The selected sequences are mapped on to the structure and the interacting amino acid residue pairs are identified. This information is converted into a normalized 20×20 matrix by the following equation:
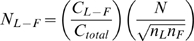
where *C_L−F_* = total number of contacts between amino acid (L) and (F),

*C_total_* = total number of contacts in dataset of selected sequences,

*n_L_* = number of amino acid (L) in protein,

and *N* = toal number of residue in protein

Three matrices for 7RSA have been produced by this method and are given in the supplementary material. Tables S1 and Table S2 represent the normalized value of contacting amino acid pairs in the middle part and tail region of random sequences; and Table S3 is for contacts from mid-portion of designed sequences.

**Figure 5:**
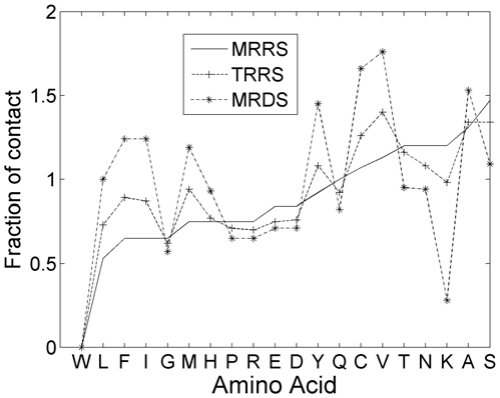
Distribution of contact of amino acids in different set of sequences. Fraction of contact made by 20 twenty different amino acids in the sets of sequences (the sequences for middle region of random sequences (MRRS), tail region of random sequences (TRRS) and middle region of designed sequences (MRDS)), generated for 7RSA. (Note: the tryptophan (W) has a value of zero since 7RSA has no tryptophan.)

The total number of interactions made by each of the 20 amino acid residues was extracted from the matrices. The fraction of contacts made by each of the amino acid type in different sets of sequences is plotted in Figure 5. (Similar pattern was obtained for other four proteins.) When we compare the contacts made by the sequences that are close the native one with completely random sequences (mid-region of the random curve), we find that the contacts made by hydrophobic residues are enhanced and the contacts of hydrophilic and polar residues have slightly decreased in the sequences close to the native. Such a behavior is enhanced in the designed sequences, with greater change than those of the random sequences close to the native one. This clearly shows that the native and the native-like (energy-wise) sequences stabilize their structure by increasing the hydrophobic interactions. By the same token, more stable sequences can be designed, as done in this case by increasing the hydrophobic contacts.

## Discussion

In the present study we have designed sequences for chosen protein conformations (Ribonuclease A (7RSA), T4-Lysozyme (1LYD), Bacillus Stearothermophilus Adenylate Kinase (1ZIP), Triosephosphate Isomerase (5TIM), and Tryptophanyl-trna Synthetase (1I6M)) on the basis of the topology based ranking [31], reduced amino acid alphabet [34] and a continuous optimization [32], [33] procedure. The results indicate that it is possible to design sequences with better energy than the native sequence in a simple and elegant manner. The fitness of these sequences in terms of their internal stability has been confirmed by comparing with a large number (80 million) of randomly generated and the designed sequences. In fact the energy distribution of the designed and randomly generated sequences has clearly been delineated. In addition, this study has provided lower and upper bounds for the energies in the sequence space.

The results have interesting biological inferences that the energy of the native sequence is at the tail end of the random distribution. It indicates that the native sequences have been minimized in the sequence space. However the optimization has not been pushed towards a global minimum, since our designed sequences perform much better than the native sequence. We can rationalize this result as follows: (i) the available energy functions mainly consider the interactions between the amino acids within the protein and the effect of environment of the protein is not adequately represented. (ii) The sequences might have evolved to attain a certain degree of stability to perform the required function. Further evolution to increase the stability may not add any advantage. This is a likely scenario since most biological systems are optimized only up to a point so that it efficiently performs the desired function.

A careful analysis of the residue-wise interaction in the designed and randomly generated sequences has shown that the native and the native-like sequences have achieved moderate stability by increasing the number of interactions between hydrophobic residues. Further increase of hydrophobic interactions has lead to high stability of designed sequences.

One can ask the question is that how relevant are these generated sequences? We have shown that the designed sequences give hits with the existing sequences in the NCBI database [41], when we constrain the position in the native sequence of some of the structurally or functionally important residue. The absence of such sequences in the database indicates that either the nature has not explored such sequences and the existing ones are a subset of total possible sequences or they are unfit for any function. It is likely that certain residues in certain positions are required for the function of the protein and the de novo design may be focused on the set of sequences which are more stable than the native ones, but in the vicinity of the native so that they retain their function.

## Materials and Methods

### (a) Generation of five-monomer sequence from the native protein

We considered five proteins from the Protein Data Bank [37]: Ribonuclease A (7RSA), T4-Lysozyme (1LYD), Bacillus Stearothermophilus Adenylate Kinase (1ZIP), Triosephosphate Isomerase (5TIM), and Tryptophanyl-trna Synthetase (1I6M), whose *C_α_* backbone conformations are shown in Figure 1.

**Figure 6:**
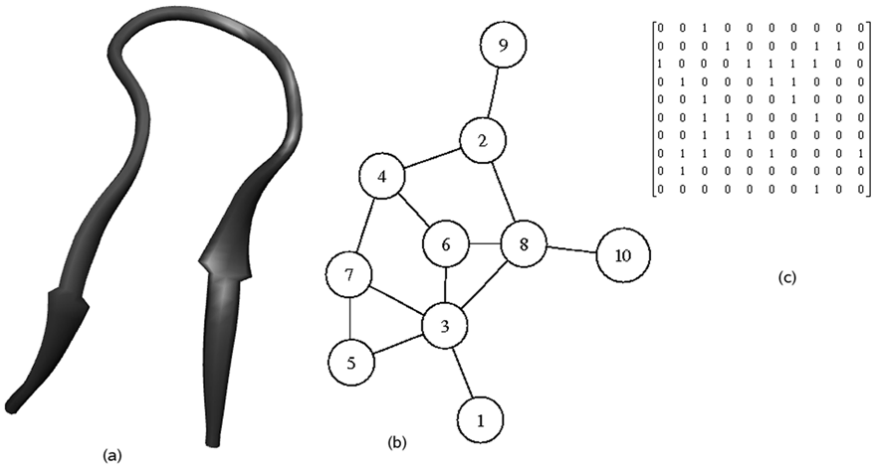
A 10-residue designed peptide CHIGNOLIN (1UAO). (a) Ribbon representation, (b) graph representation and (c) the adjacency matrix (A) for the non-covalent contacts.

We start with a protein conformation chosen from the Protein Data Bank [37] and construct its connectivity (adjacency) matrix by using its C^α^ atom coordinates. We compute the inter-residue C^α^ - C^α^ distance to construct the adjacency matrix (A) and use 6.5 Å [38] as the cut-off distance to decide the interacting pairs of residues. We exclude the adjacent residues in the chain. Thus, the element *A_ij_* = 1 if *i* ^th^ and *j* ^th^ C^α^ atoms are not the sequence neighbors and are within a distance of 6.5 Å, and 0 otherwise. This is represented by an example of a simple 10 residue peptide (Chignolin, 1UAO) in Figure 6. The molecular topology, the non-covalent connections and the corresponding adjacency matrix are given respectively in Figures 6a, 6b and 6c.

Each of the nodes (residue) in the structure graph is ranked as described in our earlier work [31]. Briefly, the nodes are weighted on the basis of their primary and secondary connections. In our earlier work, the amino acids were classified as only two types (H and P). In the present work, we have classified the amino acids into five groups labeled A, B, C, D, and E [34] as given below.

(i) A – (L F I)
(ii) B – (M V W CY)
(iii) C – (H A)
(iv) D – (T G P R Q S N E D)
(v) E – (K)

The amino acid composition in the selected protein is converted to five groups. The ranked sites are filled in the order of the types A, B, C, D, and E. (The rationale for such an assignment was discussed in Luthra et.al. [34])

This procedure is illustrated by using the example of Ribonuclease A (7RSA). The amino acid sequence of 7RSA is:

KETAAAKFERQHMDSSTSAASSSNYCNQMMKSRNLTKDRCKPVNTFVHESLADVQAVCSQKNVACKNGQTNCYQSYSTMSITDCRETGSSKYPNCAYKTTQANKHIIVACEGNPYVPVHFDASV.

According to our reduced five amino acid alphabet, 7RSA has the following composition of the five monomer types:

A = (L+F+I) = (2+3+3) = 8,

B = (M+V+W+C+Y) = (4+9+0+8+6) = 27,

C = (H+A) = (4+12) = 16,

D = (T+G+P+R+Q+S+N+E+D) = (10+3+4+4+7+15+10+5+5) = 63,

E = (K) = 10;

For 7RSA's conformation, we design a sequence with five monomer types by assigning A type to the first eight residue sites that are highly ranked. The next 27 are assigned B type and so on. Thus, we obtain the five-monomer sequence for 7RSA and, in a similar manner, for any other protein. However the total energy of the protein structure is calculated by converting the five types back into twenty monomer types as described below.

### (b) Twenty monomer sequences from a five monomer sequence

Since each of the five groups (A, B, C, D, and E) we considered have multiple amino acids, there exist numerous 20-monomer sequences corresponding to the lowest-energy five-monomer sequence. For instance, A type of monomer can be replaced by leucine, phenylalanine or isoleucine. For example, the number of possible 20-monomer sequence for 7RSA is4.2×10^87^, as computed using the information on the number of amino acids in each of the five types. Thus the number of possible designed sequences for 7RSA with the composition fixed at the type level is:
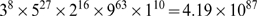



The number of sequences with the same composition of twenty amino acids as 7RSA reduces to 2.0×10^70^, as shown below.
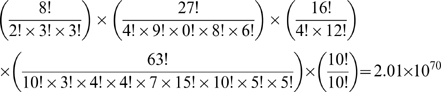



However this is a very large number for generating the sequences and we have generated a fraction of this number as described in section (d).

### (c) Evaluation of the Energy and Lower bound in the sequence space

We have computed the energies using MJ potential [38], as well as two other potentials (Kolimski and Skoinick [39] and Hinds and Levitt [40]). Since the general features were qualitatively similar, the results pertaining only to MJ potential are presented.

Global minimum energy conformation can be identified from a complete enumeration of the sequence space. However an estimate of the lower bound can be obtained by optimization techniques. We have chosen the optimization method of moving asymptotes (MMA) [42] to find possible lowest energy sequence(s) with the same amino acid composition. The energy of a sequence in the chosen conformation is evaluated by using the Minayawa-Jernigan (MJ) matrix [38], for the interacting pairs of amino acids. We have used a gradient based optimization method [33] for continuous modeling of the sequence space. The optimization problem is stated below.
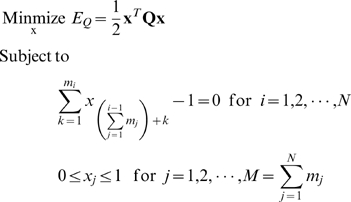
where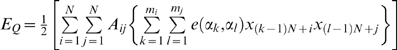 = total inter-residue energy of the protein,

**x** = an array of *M* variables that determines the type of the amino acids at each residue site,

*N* = the number of residue sites in the protein chain,

*m_i_* = the number of permitted amino acids at the *i^th^* residue site, and

**Q** = an *M*×*M* matrix that gives the total inter-residue energy as per *E_Q_*

In the above problem, we continuously vary the type of amino acid at each residue site among different amino acids within a group. Since we have five groups (A, B, C, D, and E) and have different number of amino acids in each group, *m_i_* is different for each residue site. In order to ensure that more than one amino acid does not occupy a particular site, we have used a constraint in the above equation to take care of this problem. The continuous nature of the variables in **x** enables us to vary the type of amino acid so that gradients can be computed easily. If 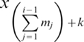 is equal to one, it means that *k* ^th^ amino acid type is assigned to *i* ^th^ residue site. In that case, the constraint ensures that the other variables associated with the *i* ^th^ residue site are zero. The formulation of this problem also permits shared occupation by different amino acids belonging to the same group at the *i* ^th^ residue site are zero. The energy function *E_Q_* is written such that it is exact when only one amino acid is assigned to a site as well as when multiple amino acids occupy the same site. This feature enables us to have a continuous modeling of the discrete sequence space.

The energy of the sequences obtained from this optimization method is shown by triangle (Δ) in Figures 2 and 4 for the five different proteins. The optimization method takes a few minutes on a single-processor P4 desktop computer in its implementation in Matlab.

### (d) Generation of Sequences

Although a complete enumeration of the sequences for a structure is not possible, we have generated a fraction of the sequence space (∼10^9^). Sequence sampling was done by considering eight different sets of sequences with different constraints and conditions. 10^8^ sequences were generated in each of the sets. The methods adopted for the generation of these eight sets are summarized in Table 1. The composition was fixed in all cases to be in agreement with the composition of the amino acids in the native sequence. In order to evaluate the performance of the designed sequences, sets of sequences (sets 5–8) which are close to the designed ones were generated. These sequences were compared with other sets of sequences (sets 1–4) which were searched randomly in the sequence space.

Sequences in set-1 were generated in a completely random fashion obeying the composition rule. The sets-2 and 3 are also random sequences in which residues in certain positions are constrained based on conservation and on the topological rank (using the node weights on the basis of their primary and secondary connections [31]). In set-4, we arbitrarily divided twenty amino acids into five types, each consisting of four residues. Then a random sequence of five types was generated. In the next step, sequences of twenty amino acids were generated from this five type sequence by randomly choosing the residues which belong to the same type (This experiment was done to see the effect of any kind of constraint).

In the designed sequence sets (set 5–8), topology based ranking scheme (using the node weights on the basis of their primary and secondary connections) [31] was used to rank the residues sites and a sequence of reduced amino acid alphabet of five groups (labeled as A, B, C, D, and E) [34] as described in section (a) was generated. Sequences in set 5 were generated by converting the five type of monomer to twenty types as described in section (b). In sets 6–8, about 10% of the amino acids were constrained in their structural position on the basis of conservation in the family of protein, component of the largest cluster (following the procedure given in Brinda and Vishveshwara [43]), and the top ranked residues [31]. The list of conserved positions and respective amino acids has been given in Table 5. The other 90% of the positions were filled as described for set-5.

**Table 5:** List of conserved residue for generation of designed sequences (set 6–8) of 7RSA.

S. No.	Conserved sequence positions from 7RSA family	Conserved amino acids in the 7RSA family	Conserved sequence positions from largest cluster [43]	Conserved amino acids from largest cluster [43]	Conserved sequence positions from top ranking [31]	Conserved amino acids from top ranking [31]
1	26	C	7	K	8	F
2	30	M	8	F	26	C
3	40	C	11	Q	27	N
4	41	K	12	H	30	M
5	44	N	35	L	31	K
6	45	T	41	K	55	Q
7	46	F	44	N	72	C
8	58	C	45	T	73	Y
9	84	C	97	Y	84	C
10	95	C	117	P	97	Y
11	97	Y	120	F	108	V
12	110	C			110	C
13	117	P				
14	118	V				
15	119	H				

In this study, we have considered five proteins of different sizes and folds (Ribonuclease A (7RSA), T4-Lysozyme (1LYD), Bacillus Stearothermophilus Adenylate Kinase (1ZIP), Triosephosphate Isomerase (5TIM), and Tryptophanyl-trna Synthetase (1I6M)). An extensive search as described above has been done on 7RSA and 1LYD. Since similar results were obtained for these proteins, only two types of sequences (10^8^) were generated for 1ZIP, 5TIM and 1I6M. The time taken by each set of sequences (10^8^) depends on the number of amino acids in the protein. For example, it took 7 days on a single-processor P4 desktop computer for each set in the case of 5TIM (249residues). In the same way the time for calculating the energy of all sequences of one set was around 75 hours.

## Supporting Information

Table S1 Contacting pairs in the middle part of random sequences (for 7RSA)
(0.03 MB DOC)

Table S2 Contacting pairs in the tail region of random sequences (for 7RSA)
(0.03 MB DOC)

Table S3 Contacting pairs in the middle part of ranked sequences (for 7RSA)
(0.03 MB DOC)
