# Supplementary material for: A Search for Energy Minimized Sequences of Proteins
Source: PLoS One. 2009 Aug 19;4(8):e6684. doi: 10.1371/journal.pone.0006684 (PMC2724685; doi:10.1371/journal.pone.0006684)
Supplement: Table S2 — Contacting pairs in the tail region of random sequences (for 7RSA) (0.03 MB DOC) [file pone.0006684.s002.doc]

Table S2: Contacting pairs in the tail region of random sequences (for 7RSA)

LEU PHE ILE MET VAL TRP CYS TYR HIS ALA THR GLY PRO ARG GLN SER ASN GLU ASP LYS

| 0.02 0.04 0.04 0.04 0.06 0.00 0.05 0.05 0.03 0.06 0.05 0.02 0.03 0.03 0.04 0.05 0.04 0.03 0.02 0.04 | 0.04 0.03 0.05 0.05 0.08 0.00 0.07 0.05 0.04 0.07 0.05 0.03 0.04 0.03 0.04 0.06 0.05 0.03 0.03 0.04 | 0.04 0.05 0.04 0.05 0.08 0.00 0.07 0.05 0.03 0.07 0.05 0.03 0.03 0.03 0.04 0.06 0.05 0.03 0.03 0.04 | 0.04 0.05 0.05 0.04 0.07 0.00 0.07 0.06 0.04 0.07 0.06 0.03 0.04 0.03 0.05 0.07 0.05 0.03 0.03 0.05 | 0.06 0.08 0.08 0.07 0.11 0.00 0.10 0.08 0.06 0.11 0.09 0.05 0.05 0.05 0.07 0.10 0.08 0.05 0.05 0.07 | 0.00 0.00 0.00 0.00 0.00 0.00 0.00 0.00 0.00 0.00 0.00 0.00 0.00 0.00 0.00 0.00 0.00 0.00 0.00 0.00 | 0.05 0.07 0.07 0.07 0.10 0.00 0.10 0.07 0.05 0.10 0.08 0.04 0.05 0.04 0.06 0.09 0.07 0.05 0.05 0.06 | 0.05 0.05 0.05 0.06 0.08 0.00 0.07 0.05 0.04 0.08 0.07 0.04 0.04 0.05 0.05 0.08 0.06 0.04 0.04 0.06 | 0.03 0.04 0.03 0.04 0.06 0.00 0.05 0.04 0.02 0.06 0.05 0.03 0.03 0.03 0.04 0.06 0.05 0.03 0.04 0.04 | 0.06 0.07 0.07 0.07 0.11 0.00 0.10 0.08 0.06 0.09 0.09 0.05 0.05 0.05 0.07 0.10 0.08 0.05 0.05 0.07 | 0.05 0.05 0.05 0.06 0.09 0.00 0.08 0.07 0.05 0.09 0.07 0.04 0.05 0.04 0.06 0.09 0.07 0.05 0.05 0.06 | 0.02 0.03 0.03 0.03 0.05 0.00 0.04 0.04 0.03 0.05 0.04 0.01 0.02 0.02 0.03 0.05 0.04 0.02 0.03 0.03 | 0.03 0.04 0.03 0.04 0.05 0.00 0.05 0.04 0.03 0.05 0.05 0.02 0.02 0.03 0.04 0.05 0.04 0.03 0.03 0.04 | 0.03 0.03 0.03 0.03 0.05 0.00 0.04 0.05 0.03 0.05 0.04 0.02 0.03 0.02 0.04 0.06 0.04 0.04 0.03 0.03 | 0.04 0.04 0.04 0.05 0.07 0.00 0.06 0.05 0.04 0.07 0.06 0.03 0.04 0.04 0.04 0.07 0.06 0.04 0.04 0.05 | 0.05 0.06 0.06 0.07 0.10 0.00 0.09 0.08 0.06 0.10 0.09 0.05 0.05 0.06 0.07 0.10 0.08 0.06 0.06 0.07 | 0.04 0.05 0.05 0.05 0.08 0.00 0.07 0.06 0.05 0.08 0.07 0.04 0.04 0.04 0.06 0.08 0.06 0.05 0.05 0.06 | 0.03 0.03 0.03 0.03 0.05 0.00 0.05 0.04 0.03 0.05 0.05 0.02 0.03 0.04 0.04 0.06 0.05 0.02 0.04 0.05 | 0.02 0.03 0.03 0.03 0.05 0.00 0.05 0.04 0.04 0.05 0.05 0.03 0.03 0.03 0.04 0.06 0.05 0.04 0.03 0.05 | 0.04 0.04 0.04 0.05 0.07 0.00 0.06 0.06 0.04 0.07 0.06 0.03 0.04 0.03 0.05 0.07 0.06 0.05 0.05 0.04 |
| --- | --- | --- | --- | --- | --- | --- | --- | --- | --- | --- | --- | --- | --- | --- | --- | --- | --- | --- | --- |
