# Supplementary material for: A Search for Energy Minimized Sequences of Proteins
Source: PLoS One. 2009 Aug 19;4(8):e6684. doi: 10.1371/journal.pone.0006684 (PMC2724685; doi:10.1371/journal.pone.0006684)
Supplement: Table S3 — Contacting pairs in the middle part of ranked sequences (for 7RSA) (0.03 MB DOC) [file pone.0006684.s003.doc]

Table S3: Contacting pairs in the middle part of ranked sequences (for 7RSA)

LEU PHE ILE MET VAL TRP CYS TYR HIS ALA THR GLY PRO ARG GLN SER ASN GLU ASP LYS

| 0.02 0.09 0.09 0.08 0.14 0.00 0.13 0.10 0.05 0.07 0.03 0.02 0.02 0.02 0.03 0.03 0.03 0.02 0.02 0.00 | 0.09 0.07 0.13 0.08 0.18 0.00 0.16 0.12 0.05 0.08 0.04 0.02 0.03 0.03 0.03 0.04 0.04 0.03 0.03 0.00 | 0.09 0.13 0.07 0.08 0.18 0.00 0.16 0.12 0.05 0.08 0.04 0.02 0.03 0.03 0.03 0.04 0.04 0.03 0.03 0.00 | 0.08 0.08 0.08 0.08 0.08 0.00 0.09 0.09 0.09 0.08 0.05 0.05 0.05 0.05 0.05 0.05 0.05 0.05 0.05 0.01 | 0.14 0.18 0.18 0.08 0.13 0.00 0.10 0.09 0.11 0.16 0.08 0.05 0.05 0.05 0.06 0.10 0.08 0.06 0.06 0.01 | 0.00 0.00 0.00 0.00 0.00 0.00 0.00 0.00 0.00 0.00 0.00 0.00 0.00 0.00 0.00 0.00 0.00 0.00 0.00 0.00 | 0.13 0.16 0.16 0.09 0.10 0.00 0.11 0.09 0.10 0.14 0.07 0.05 0.05 0.05 0.06 0.10 0.07 0.06 0.06 0.01 | 0.10 0.12 0.12 0.09 0.09 0.00 0.09 0.10 0.10 0.11 0.06 0.05 0.05 0.05 0.06 0.07 0.06 0.05 0.05 0.02 | 0.05 0.05 0.05 0.09 0.11 0.00 0.10 0.10 0.05 0.07 0.03 0.03 0.03 0.03 0.03 0.03 0.03 0.03 0.03 0.00 | 0.07 0.08 0.08 0.08 0.16 0.00 0.14 0.11 0.07 0.20 0.08 0.02 0.03 0.03 0.05 0.16 0.08 0.03 0.03 0.02 | 0.03 0.04 0.04 0.05 0.08 0.00 0.07 0.06 0.03 0.08 0.07 0.03 0.04 0.04 0.06 0.07 0.07 0.04 0.04 0.03 | 0.02 0.02 0.02 0.05 0.05 0.00 0.05 0.05 0.03 0.02 0.03 0.02 0.03 0.03 0.03 0.03 0.03 0.03 0.03 0.02 | 0.02 0.03 0.03 0.05 0.05 0.00 0.05 0.05 0.03 0.03 0.04 0.03 0.03 0.03 0.04 0.03 0.04 0.03 0.03 0.02 | 0.02 0.03 0.03 0.05 0.05 0.00 0.05 0.05 0.03 0.03 0.04 0.03 0.03 0.03 0.04 0.03 0.04 0.03 0.03 0.02 | 0.03 0.03 0.03 0.05 0.06 0.00 0.06 0.06 0.03 0.05 0.06 0.03 0.04 0.04 0.05 0.05 0.06 0.04 0.04 0.02 | 0.03 0.04 0.04 0.05 0.10 0.00 0.10 0.07 0.03 0.16 0.07 0.03 0.03 0.03 0.05 0.08 0.07 0.04 0.04 0.04 | 0.03 0.04 0.04 0.05 0.08 0.00 0.07 0.06 0.03 0.08 0.07 0.03 0.04 0.04 0.06 0.07 0.07 0.04 0.04 0.03 | 0.02 0.03 0.03 0.05 0.06 0.00 0.06 0.05 0.03 0.03 0.04 0.03 0.03 0.03 0.04 0.04 0.04 0.04 0.04 0.02 | 0.02 0.03 0.03 0.05 0.06 0.00 0.06 0.05 0.03 0.03 0.04 0.03 0.03 0.03 0.04 0.04 0.04 0.04 0.04 0.02 | 0.00 0.00 0.00 0.01 0.01 0.00 0.01 0.02 0.00 0.02 0.03 0.02 0.02 0.02 0.02 0.04 0.03 0.02 0.02 0.00 |
| --- | --- | --- | --- | --- | --- | --- | --- | --- | --- | --- | --- | --- | --- | --- | --- | --- | --- | --- | --- |
